# Therapeutically Harnessing Tumor Cell-Derived Extracellular Vesicles for Multiple Myeloma: Recent Advances and Future Perspectives

**DOI:** 10.3390/pharmaceutics16111439

**Published:** 2024-11-11

**Authors:** Shumei Xiao, Lei Chen, Zhichao Chen, Qiubai Li

**Affiliations:** 1 Department of Rheumatology and Immunology, Union Hospital, Tongji Medical College, Huazhong University of Science and Technology, Wuhan 430022, China; xsmncdx2018@163.com; 2 Department of Hematology, Union Hospital, Tongji Medical College, Huazhong University of Science and Technology, Wuhan 430022, China; lene918@163.com (L.C.); chenzhichao@hust.edu.cn (Z.C.); 3 Hubei Engineering Research Center for Application of Extracellular Vesicles, Hubei University of Science and Technology, Xianning 437100, China

**Keywords:** multiple myeloma, extracellular vesicles, biomolecular cargo, diagnosis, therapeutic drug

## Abstract

Extracellular vesicles (EVs) have emerged as pivotal regulators for extensive intercellular crosstalk owing to capsuled diverse bioactive substances such as proteins, nucleic acids, and lipids. Recent studies have shown that tumor-derived EVs significantly influence the bone marrow microenvironment, contributing to the progression of multiple myeloma (MM). This highlights the robust potential of EVs as a promising avenue for developing more effective and precise diagnostic and therapeutic strategies for MM. In this review, we briefly discuss the multifaceted roles of EVs in MM progression, as well as the diagnostic and therapeutic value in MM management. Specifically, we focus on the latest research progress regarding the therapeutic potential of EVs for MM, particularly tumor cell-derived EVs, as we elaborate on three main aspects: (i) EVs as therapeutic targets, including the targeted inhibition of EV biogenesis and uptake, and the possibility of eliminating tumor-derived EVs; (ii) EVs as delivery nanovectors, where we discuss the latest anti-MM candidates and potential ways to optimize therapeutic efficiency; and (iii) engineered EVs as antitumor vaccines, focusing on the use of tumor cell-derived EVs in immunotherapy. Finally, we address the prospects and challenges of harnessing the therapeutic potential of EVs in clinical transformation.

## 1. Introduction

Multiple myeloma (MM) is recognized as the second most prevalent hematologic malignancy, caused by abnormal monoclonal growth of plasma cells in the bone marrow (BM) with an excessive expression of the M component [1]. Patients with MM frequently experience a range of typical clinical manifestations associated with organ damage, including bone pain and fractures, anemia, infections, renal failure, and hyperviscosity [1,2]. Of note, MM patients almost always evolve from monoclonal gammopathy of undetermined significance (MGUS), a premalignant stage without obvious symptoms, with or without a more advanced intermediate stage termed as smoldering MM (SMM) [3]. The advancement of new and targeted regimens for MM therapy, including proteasome inhibitors, immunomodulators, and monoclonal antibodies (CD38 monoclonal antibody, etc.) as well as autologous stem cell transplantation (ASCT), has effectively improved patient outcomes and prolonged MM survival [4]. However, drug resistance, high relapse rates, and dynamic adverse effects accompanying current treatments still compromise the efficacy of existing therapeutic options [5]. Therefore, based on explorations of in-depth mechanisms for MM biology, other novel and practical strategies are needed to achieve better prognosis and clinical management for MM patients.

Extracellular vesicles (EVs) are ubiquitous nanoscale vesicles in the extracellular matrix secreted by almost all cells. Conventionally, EVs can be classified into three main categories based on the dissection of biogenesis, comprising exosomes generated via the endocytosis pathway, microvesicles (MVs) directly formed by plasma membrane outward budding, and apoptotic vesicles released from apoptotic cells. However, there are no definitive approaches and molecular markers to trace the EVs’ biogenesis pathways, and it is recommended to operationally classify EVs according to their biophysical or biochemical features [6]. In this review, we adopt the terminologies that the authors used to illuminate their discoveries with the collective term “EVs” when undefined. At the earliest time, EVs were originally thought to be cellular debris for cellular waste product removal, while abundant evidence has corroborated their function as an important mode of intercellular transport systems and interactions [7,8]. The cargo molecules contained in EVs include abundant RNA, proteins, lipids, and metabolites, along with other parental bioactive substances, which are selectively packaged through the complex endosomal or endosome-assisted biogenesis pathway and could be transported into the recipient cells, consequently influencing their biological properties [9].

Accumulating evidence highlights that EVs are robust communicators for cell-to-cell crosstalk in the tumor microenvironment. Indeed, tumor-derived EVs (TDEVs) extensively participate in the complex processes of cancer occurrence and progression via abundant bioactive substances inherited from parental tumor cells. Accordingly, the intrinsic involvement of EVs derived from MM cells (MM-EVs) in various tumor progression hallmarks has provoked strong attention, including tumor growth, angiogenesis, osteolysis, immunosuppression, drug resistance, etc. This review presents a brief overview of the multifaceted role of TDEVs in MM biology, describes their clinical value as efficient markers, and mainly highlights the updated discoveries on the potential prospects of EV-based therapy for MM.

## 2. Overview of Functional Roles of EVs in MM

### 2.1. Biological Regulation of MM Cells

**Figure 1:**
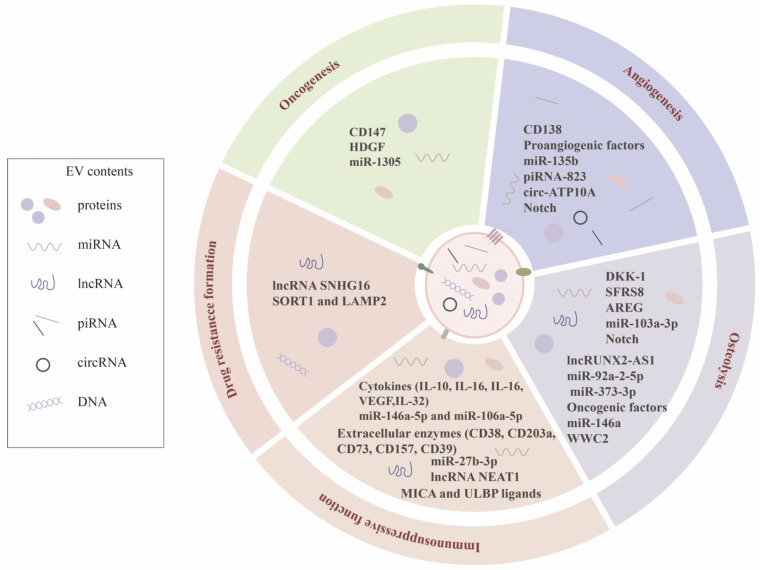
Roles of TDEVs in MM biology. In MM, TDEVs contain a lot of bioactive molecules, including protein, miRNA, lncRNA, piRNA, circRNA, and dsDNA. Therefore, TDEVs are significant communicators for both MM cell autocrine effects to promote tumor cell proliferation and drug resistance formation, and tumor–nontumor communication contributing to angiogenesis, osteolysis, and immunosuppression.

Molecular insight into the cargo of MM-EVs has unraveled the crucial autocrine and paracrine function involved in MM biology (Figure 1). It is well known that MM-EVs could function on tumor cells to promote neoplastic growth, stemness maintenance, and drug-resistance formation [10,11,12]. For instance, enriched hepatocyte-derived growth factor (HDGF) in MM-EVs could be transferred into MM cells to promote proliferation and inhibit mitochondrial respiration via the AKT pathway [13]. MM side population cells with CSC-like properties transferred lncRNA SNHG16 through exosomes thus causing dexamethasone resistance in main population cells [14]. More recently, a study reported that lenalidomide-resistant MM cells are capable of enhancing cell adhesion and EV production thus causing DR in sensitive MM cells, possibly being inhibited by the silence of SORT1 and LAMP2 genes associated with EV secretion [15]. In addition, MM-EVs could also favor extracellular environment inflammation to promote MM cell viability and migration, via stimulating BM microenvironment cells to release more cytokines, chemokines, and proteases, like IL-6, IL-8, CXCL1, matrix metallopeptidase 9 (MMP-9), and so on [16,17].

### 2.2. Angionesis

MM-EVs have been proven to directly act on ECs to affect angiogenesis, proliferation, and metastasis, which is associated with multiple cargos, like CD138 [18], proangiogenic factors [19,20], miR-135b [21], and piRNA-823 [22]. Recently, it was demonstrated that increased serum exosomal circ-ATP10A can enhance angiogenesis by functioning as a miRNA sponge to modulate their downstream mRNAs of angiogenic cytokines, such as VEGFB, HIF1A, PDGF, and FGF [23]. Interestingly, the treatment of MM cells with bortezomib might increase the generation of EVs but limit their pro-angiogenic activity [20,24]. As a unique short-chain cell-permeable analog, the C6 ceramide (C6-cer) treatment of human OPM2 cells stimulated the secretion of exosomes and upregulated the expression of exosomal miRNAs sufficient to inhibit tumor development, including miR-202, miR-16, miR-15a, and miR-29b [25]. Furthermore, augmented exosomal miR-29b after C6-cer treatment could block the PI3K-Akt pathway in ECs to suppress angiogenesis [26].

### 2.3. Osteolysis

A multitude of evidence has highlighted the involvement of MM-EVs in the functional disequilibrium of bone-forming osteoblasts (OBs) and bone-resorbing osteoclasts (OCs), featured by facilitating the resorptive activity of OCs while inhibiting OBs’ function [27,28,29]. For instance, Zhang et al. [30] reported that MM cells released splicing factor arginine/serine-rich 8 (SFRS8) through exosomes to stimulate OCs’ malignant differentiation via facilitating the alternative splicing of the calcyclin-binding protein (CACYBP) independent of the Wnt/β-catenin pathway, meanwhile augmenting bone lesion formation in the SCID/NOD-TIBIA mouse model in vivo. AREG-enriched MM exosomes increased the secretion of pro-osteoclastogenic cytokine IL-8 in OCs and inhibited recipient MSC differentiation towards OBs, consequently resulting in the destruction of a functional balance between OBs and OCs [31].

More pertinently, the transportation of various miRNAs by MM-EVs into recipient MSCs might also be ascribed to the osteo-inhibitory function. Our group demonstrated that MM-MVs were highly presented with miRNAs negatively regulating osteogenesis together with selectively expressing key regulators of MBD, including DKK1, IL-7, and sFRP2 [11]. Additionally, Li et al. [32] showed that MM cell-derived exosomes delivered lncRUNX2-AS1 to MSCs thereby inhibiting the expression of RUNX2 to reduce the osteogenic potential of MSCs. More recently, a study demonstrated that the N6-methyladenosine (m6A) modification of hnRNPA2B1 in MM cells altered the associated miRNA processing thus leading to a high level of miR-92a-2-5p and miR-373-3p in their secreted exosomes, which enhanced osteoclastogenesis and suppressed osteoblastogenesis through blocking IRF8 or RUNX2 after being transferred into recipient monocytes or MSCs [33]. Remarkably, MM-EVs could also serve as inducers of phenotypic and functional alternations in MSCs, consequently providing a supportive niche for MM progression. For instance, a study reported that, respectively, cocultured MSCs with EVs isolated from MM cell culture supernatants and the plasma of MM patients induced MSC proliferation, while the abundant oncogenic factors (MYH4, CD166, CD44, ANXA2, and FN1, etc.) in plasma EVs could contribute to enhancing migration and adhesion [34]. MM exosomes upregulated miR-146a levels in MSCs consequently augmenting cytokines and chemokines production for MM progression through the endogenous Notch pathway [16]. Additionally, MM exosomal miR-146a was also shown to induce MSC transversion into cancer-associated fibroblasts (CAFs) with overexpressing inflammatory cytokines [35]. Apart from triggering transformation, MM-EVs also act as a vital regulator for CAF activation. MM exosomes with elevated levels of miR-27b-3p and miR-214-3p in MGUS fibroblasts were induced by transferring WWC2 protein in MM-exosomes through regulating the Hippo pathway, which prompted the proliferation and apoptosis resistance of MM fibroblasts by targeting the FBXW7 and PTEN/Akt/GSK3 pathways, respectively [36].

### 2.4. Immunosuppression

MM exosomes promoted MDSCs’ proliferation and positively regulated immunosuppressive molecules corresponding with complex signaling pathways, which might be mediated by the carried cytokines such as IL-10 and IL-16, VEGF [19,37], and miRNAs, like miR-146a-5p and miR-106a-5p [38]. In addition, MM-EVs also exert direct effects on T-cells, hence supporting immune escape. It has been suggested that T-cell suppression was directly modulated by MM exosomes via both inhibiting CD4+ T cell viability and CD8+ T cell cytotoxicity and facilitating Treg cell proliferation [39]. Intriguingly, EVs derived from the MM cell line MOPC315.BM (MOPC315.BM-derived EVs) upregulated the expression of CTLA-4 and IC PD-1 on CD4+ T cells and decreased the expression of CD27, supporting the formation of a BM immunosuppressive niche [40]. From the mechanical point of view, the immunosuppressive dysfunction of T cells resulting from MM-EVs is involved with the incorporated effector molecules. For example, extracellular enzymes such as CD38, CD203a, CD73, CD157, and CD39, contained in plasma MVs, were important for adenosine (ADO) production to suppress T-cell function [41]. MM exosomal miR-27b-3p reinforced immunosuppressive effects by downregulating CD28 expression [42]. Moreover, MM-EVs were confirmed to directly drive macrophages to M2-phenotype polarization and upregulate PD-L1 expression, mechanically associated with IL-6 activated stat3-mediated signaling pathways [43] and the IL-32-dependent PFKFB3-JAK1 axis [44], subsequently depleting the function of CD8+T cells.

However, how tumor-derived EVs modulate the immunoregulatory functions mediated by NK cells remains a matter of debate. It is believed that the molecules harbored in vesicles determine their ability to promote or inhibit NK cell activities, including cytolytic function, cytokine production, degranulation, etc. [45]. In accordance with the critical immune-inhibitory role of MM-EVs, MM exosomes have been shown to attenuate the cytotoxic function of NK cells, via significantly decreasing surface NKG2D expression [46,47]. It was proposed that elevated lncRNA NEAT1 in MM exosomes accounted for the mediated inhibitory effect on NK cells through the EZH2/PBX1 axis [47]. In-depth, the NKG2D- NKG2D ligand (NKG2DL) pathway is a significant mode associated with the recognition and elimination of MM cells mediated by NK cells [48]. In this scenario, Vulpis et al. [49,50] provided new insights into the dual action of MM-EVs’ mediated delivery of NKG2DLs represented by MIC and ULBP molecules on the NK cell function. They found that MICA*008 (MICA allelic variant) could be exposed on the surface of MICA*008 transfected MM cell-derived exosomes and MVs, which was able to augment the tumoricidal function of NK cells for a short time, but their prolonged stimulation downregulated NKG2D to inhibit NK cell function [49]. Meanwhile, NK cells could be induced to fratricide due to the transport of MICA*008 mediated by MM-EVs, revealing a novel mechanism of impairing the NK cell response thus favoring immune escape and tumor progression [49]. Interestingly, they further revealed that the cross-dressing on MM cells could be manipulated by the delivery and diffusion of MICA and ULBP ligands via MVs, consequently augmenting the capability of MM cells to trigger NK cell cytotoxicity [50].

### 2.5. As a Senescence-Associated Secretory Phenotype (SASP)

Crucially, under various senescent stressors or natural aging, increased EVs are found to function as emerging constituents of SASP, known as senescence-associated EVs [51]. These EVs then send destructive signals to cellular recipients, thus destroying protein networks and energy and culminating in senescence pattern transmission [51]. This expands the field to investigate the complex role of senescence in age-associated diseases, like cancer [52]. Since aging is a favorable risk factor of MGUS and MM, we speculate that an in-depth understanding of senescence-associated EVs could render insights into aging on the MM pathogenesis and therapeutic resistance, inspiring a new path forward for MM therapy via senescence-based anticancer therapies.

On the one hand, cancer cells can be led to senescence in response to chemotherapeutic agents, which is called therapy-induced senescence (TIS). It is evident that TIS cells secrete significantly more EVs but their consequence in the tumor environment is controversial. Borrolli and colleagues previously confirmed that low doses of genotoxic drugs including doxorubicin (DOX) and melphalan (MEL) induced an SASP phenotype in MM cells and the senescent MM cells could stimulate NK cells degranulating and releasing IFN-γ by up-expressing NK cell-activating ligands [53,54]. They discovered that MEL-induced senescent MM cells released more exosomes with the expression of IL15RA and IL15, in addition to promoting NK cell proliferation and activation in the presence of exogenous IL15 [53]. Recently, their group further reported that DOX-treated MM cells increased the enrichment of miR-433 into exosomes, which in turn reinforced a senescent-like phenotype on bystander senescence through miR-433-driven CDK-6 downregulation without triggering DNA-damage response [54]. The above evidence implies that senescence-associated EVs might serve as an initial anticancer mechanism through arresting nearby tumor cells and fostering immune surveillance. However, it has been suggested that that EVs from senescent tumor cells can function as pro-tumorigenic SASPs [55]. For example, Kavanagh et al. investigated the protein and chemotherapy content of EVs derived from TIS triple negative breast cancer cells and found that the senescence-associated EVs contained chemotherapy and key proteins involved in cell proliferation, ATP depletion, and apoptosis [56]. In MM, the accumulation of senescent MSCs in a BM microenvironment is a pivotal factor supporting myeloma progression [57]. Previous reports have shown that MM cells induced senescence MSCs by inhibiting Dicer1 expression and modulating the microRNA profile, which impeded their differentiation potential and in turn promoted MM growth [58,59]. Additionally, it has been reported that MM cell upregulated the expression of SASP genes (eg. IL6, IL8, CXCL1, and CXCL2) and disrupted normal metabolism in bone marrow adipocytes, causing bone damage and drug resistance formation [60]. Although these studies did not characterize whether stromal cell senescence is driven by paracrine senescence mediated by EVs, considering that EVs act as signaling molecules mediating bidirectional communication, it could be speculated that MM-EVs serve as representative SASP factors of senescent tumor cells and can be more detrimental in the long term [61].

## 3. EVs as Biomarkers

In particular, EVs are promising biomarkers for noninvasive liquid biopsy owing to their features of having stable membranes, being widely distributed in various body fluids, and containing abundant tumor-derived materials, as well as being detected in a minimally invasive manner [62]. In MM, a plethora of evidence emphasized the exciting opportunities of EVs as critical substances to find more efficient biomarkers, meanwhile helping to further inform clinical treatments (Table 1).

Commonly, it has been suggested that the number of EVs, especially those containing tumor-specific phenotypes, could reflect the progression and tumor burden fluctuation of MM, beneficial for diagnosis [63,64,65]. For instance, Krishnan et al. [65] revealed that high levels of CD138+ MVs implied significant prognostic value in predicting “relapse risk” and treatment response. Later, they expanded the findings on several markers including P-gp/PS/CD138/CD34, further linking the potential relationship between circulating large EVs (specifically MVs) and disease burden and progression, as well as multidrug-resistance formation [66]. Likewise, our team also found that the higher concentration of circulating CD138+ MVs indicated more severe MBD and renal impairment (RI) in MM patients [11,67]. Furthermore, a study comprehensively investigated the association between increased circulating CD38+, CD138+, and CD38+/CD138+ EVs and MM clinical parameters [68]. In particular, CD38+/138+EVs were positively correlated with the BM-PC percentage and sFLCkappa level [68]. More recently, Liu et al. [69] reported that Ps+CD41a−, Ps+CD41a−CD138+, and Ps+CD41a−BCMA+ MVs are predictive markers correlated with MM burden, among which, Ps+CD41a−MVs is considered as a potential index for the measurable residual disease (MRD) test. Of note, it has been identified that PB-EVs could be used for MRD monitoring via indicating disease markers in the BM [70,71,72]. In particular, the absence of MM markers, including CD38, CD56, CD117, and CD27, in PB-EVs from HD could also be helpful for MRD monitoring [71]. What is more, it was also observed that PD-L1 expression on the EVs was positively associated with the response to DARA treatment in patients [70]. Intriguingly, a real-world study reported that a high EV protein/particle ratio (>0.6 µg/10^8^ articles) was associated with poorer survival and prognostic features including immune dysfunction, sFLC, and the duration of the treatment response [72]. More surprisingly, Lia et al. [73] explored the potential relevance of surficial antigens on EVs to acute GVHD, indicating that CD146 was a higher risk factor for GVHD development, while CD31 and CD140-α were indicative of a reduced risk.

Despite the counts and phenotype of EVs, the distinct molecular profiles of TDEVs can be used as markers in MM clinical practice. Enriched CD44 in serum EVs was potentially associated with the prediction of overall survival [74]. Of note, advanced technological developments have offered a comprehensive insight into the differences in composition and functionality of protein cargos in various EV subpopulations, especially large and small extracellular vesicles, as well as their size distribution, thus underling the necessity for reconsidering the heterogeneous potential of EV proteins based on EV subtypes [75,76,77]. In MM, a recent study highlighted that higher CD71 in SEVs and LEVs from patients of MM vs. MGUS is a significant diagnostic and prognostic biomarker, and that increased CD40 in LEVs derived from MM patients is positively associated with standard and high-risk cytogenetics [78]. However, concerning the variations in approaches for isolation, the complicated cultivation contexts, and different cell lines, it is still very difficult to achieve a conclusive understanding of the protein profile in different EV subpopulations [79].

As for RNA cargo, miRNA has the most abundant research data. Exosomal let-7b, let-7e, miR-106a, miR-106b, miR-155, miR-16, miR-17, miR-18a, and miR-20a were considered to be predominant predictors for progression-free survival (PFS), while let-7b and miR-18a were used to predict overall survival (OS) [80]. Furthermore, exosomal miRNAs (let-7d-5p/miR-425a-5p/miR-185-5p/let-7c-5p/miR-140-3p) were also preferable markers correlated with disease progression, β2-microglobulin, and plasma cell load [81,82]. In addition, a comparison of miRNA, mRNA, and lncRNA profiles in serum EVs in patients with different responses to chemotherapy demonstrated the potential of EVs as a marker related to chemotherapy sensitivity and a predictive marker of drug resistance in MM patients [82,83]. Sedlarikova et al. [84] reported that only one abnormal exosomal lncRNA PRINS could differentiate MM and MGUS patients from HD with a sensitivity of 84.9% and a specificity of 83.3%. Luo et al. [85] found that higher levels of circMYC in exosomes were positively correlated with 17p deletion and t(4;14) translocation, D-S staging, and ISS staging, serving as a predictor of poor prognosis and recurrence risk and mortality for MM patients. In addition, exosomal circRNA, chr2:2,744,228–2,744,407 + was upregulated, thus inducing MM-related peripheral neuropathy (PN) via the downstream hsa-miR-6829-3p/GRIN2B axis, which could be associated with the clinical characteristics and prognosis of PN [86]. Recently, Sun et al. [87] demonstrated that exosomal circ-G042080 was positively correlated with MM progression and MM-associated myocardial injury. Particularly, Kim et al. [88] dissected the selective enrichment patterns of cell-free messenger RNA (cf-mRNA) in EVs and non-vesicular particle fractions and further investigated the differential gene expression mode in EVs to explore the potential diagnostic role of vesicular cf-mRNA. It was found that distinctive differentiating mRNAs carried by MM-EVs corresponded to toxic substance detoxification compared with healthy individuals. Moreover, Wu et al. [89] found that the mRNA level of the receptor for glycation end products (RAGE) in serum EVs obtained from MM patients with sarcopenia was elevated. Mechanically, RAGE carried in mouse MM-EVs could trigger the TLR4/NF-κB p65 pathway in C2C12 cells to facilitate inflammatory responses, ROS expression, and myotube atrophy in vivo and in vitro.

Moreover, EVs are also a valuable trove of DNA, which is mostly double-stranded, including mutated and amplified oncogenes and transposable elements that may reflect the mutational status of the entire genome and parental tumor cells [90,91]. Compared with cell-free DNA (cf-DNA), dsDNA in EVs can serve as an alternative cancer-derived genomic material with better preservation and concentration, and has been found to be superior to cf-DNA in several studies of mutation detection in solid malignancies [92]. However, the same superiority of EV-DNA in MM detection was not confirmed in the only currently available report that targeted sequencing results in EV-DNA and comprehensively compared the five different DNA sources (BM-DNA, cfDNA, CTC-DNA, PBMNCs-DNA, and EV-DNA) [93]. The possible reasons for the inferior outcome might be the differences in EV-DNA extraction, isolation, and characterization protocols.

**Table 1:** Summary of the evidence for EV biomarkers in MM.

Origin	EV Size	Potential Biomarker	Clinical Application	References
Serum	40–120 nm	Concentration	Diagnosis	[63]
Serum	<300 nm	CD38	Diagnosis	[64]
Platelet-free plasma (PFP)	0.1–1 μm	CD138	Diagnosis and prognosis	[65]
PFP	—	CD138/P-gp/PS/CD34	Diagnosis	[66]
Plasma	200–1000 nm	CD138	Diagnosis	[11]
Plasma	10–1000 nm	CD138	Diagnosis	[67]
Serum	20–800 nm	CD138/CD38	Diagnosis and prognosis	[68]
Bone marrow (BM)	—	Ps/BCMA/CD138	Diagnosis and prognosis	[69]
PB and BM	—	CD147/CD55/CD59/ PD-L1	Diagnosis and prognosis	[70]
PB and BM	<150 nm	EV protein/particle ratio (EVc)	Prognosis	[72]
Serum	—	CD146/CD31/CD140-α	Diagnosis and prognosis	[73]
Serum	—	CD44	Diagnosis and prognosis	[74]
PFP	small EVs (SEVs) and large EVs (LEVs)	CD71	Diagnosis and prognosis	[78]
		CD40		
Plasma	<120 nm	Let-7b, let-7e, miR-106a, miR-106b, miR-155, miR-16, miR-17, miR-18a, and miR-20a	Prognosis	[80]
		Let-7b, miR-18a	Prognosis	
Serum	50–60 nm	Let-7d-5p, miR-425a-5p	Diagnosis and prognosis	[81]
		Let-7c-5p, miR-140-3p, miR-185-5p, and miR-425-5p	Prognosis	
Plasma	<100 nm	MiR-16, miR-15a, miR-20a and miR-17	Predict chemoresistance	[82]
Serum	100–150 nm	482 lncRNAs and 2099 mRNAs	Predict chemoresistance	[83]
Serum	—	LncRNA PRINS	Diagnosis	[84]
Serum	—	CircMYC	Diagnosis and prognosis	[85]
Serum	<100 nm	CircRNA, chr2:2,744,228–2,744,407 +	Diagnosis	[86]
Serum	<120 nm	Circ-G042080	Predict complications	[87]
Plasma	30–100 nm	Selective enrichment pattern of cf-mRNA	Diagnosis	[88]
Serum	<100 nm	RAGE mRNA	Predict complications	[89]

## 4. EVs as Therapeutic Targets

**Figure 2:**
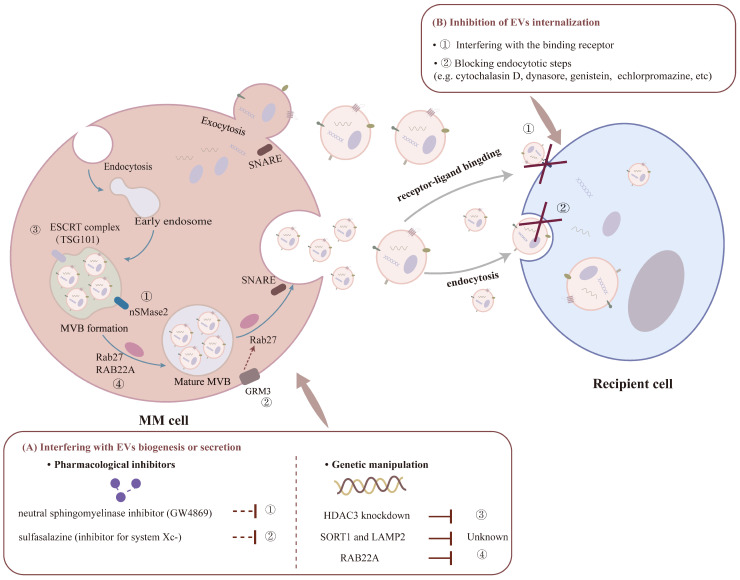
EVs as therapeutic targets in MM. TDEVs can be targeted to decrease their deleterious effects of inhibition by main two approaches: interfering with EVs biogenesis or secretion by pharmacological inhibitors and genetic manipulation to regulate the target molecules associated with EV formation, like nSMase2, Rab 27, and RAB22A; inhibiting MM-EVs internalization into recipient cells by interfering with the binding receptor or blocking endocytosis.

Numerous studies have emphasized the orchestral roles of EVs in intercellular crosstalk of tumor biology. In order to decrease the deleterious effects of EVs, strategies for therapeutic purposes have been designed based on the targeted inhibition or clearance of these EVs, which can be achieved by the following approaches: interfering with EV biogenesis or secretion, blocking EVs’ uptake by targeted recipient cells, or diminishing the circulating EVs [94,95]. In this section, we will discuss the associated findings that could be valuable for MM therapy (Figure 2).

### 4.1. Inhibiting EV Biogenesis or Secretion

Available evidence suggests that exosomes and microvesicles are produced at different sites in distinct biosynthesis patterns but might share common intracellular mechanisms and sorting mechanisms. There is an increasing body of reviews that has extensively discussed the detailed formation and release of EVs [96,97,98]. Since the biogenesis and secretion of EVs are involved with complex mechanisms and the synergistic interactions of different molecules, whether or not different mechanisms act simultaneously, concurrently, or sequentially in a single cell is not fully understood. Therefore, how to specifically regulate the formation of EV derived from a certain kind of cell is still a major obstacle. To address this, in-depth insight into both the distinct generation mechanism and various effects mediated by bioactive cargos of TDEVs is fundamental for designing helpful interventions. Basically, one of the major recognized mechanisms of exosome formation begins with the inward budding of endosomes to form intraluminal vesicles (ILVs), which then mature into multivesicular vesicles (MVBs), which are subsequently released from the parental cells into the microenvironment after fusion of the MVBs with the plasma membrane. This intricate process requires the coordination of multiple proteins, represented by the endosomal sorting complexes required for transport proteins (ESCRT-dependent), tetrapantetin family proteins, neutral sphingomyelinase (ESCRT-independent), Rab GTPases, and soluble N-ethylmaleimide-sensitive factor attachment protein receptors (SNAREs), etc. [96]. It has been suggested that the aberrant expression of universal regulators (like TSG101, Rab 27, nSMase2, etc.) is a major cause of tumor cells secreting more EVs [94]. Accordingly, pharmacological agents, antibodies, and genetic manipulations based on the above targets have been explored to arrest EV generation [94,99].

Among this, GW4869 is a widely used neutral sphingomyelinase inhibitor for blocking ceramide-mediated inward budding of MVBs and the subsequent release of ILVs [96]. GW4869 could exert an effective anti-tumor effect by reducing EV production and affecting cargo sorting [96]. In MM, Faict et al. [29] demonstrated that GW4869 decreased exosome secretion from 5TGM1 cells thus abrogating the inhibition of OB viability mediated by the exosomes in vitro, while the application of GW4869 combined with BTZ in a 5TGM1 mouse model decreased tumor growth and micro-vessel density, with a protective function on cortical bone volume. In addition, Rab GTPases, including Rab2b, Rab9a, Rab5a, and Rab27a/b, have also been targeted to inhibit EV biogenesis, as they are fundamental for vesicular trafficking and formation [96]. For instance, Rab27a/b knockout with RNA interference (RNAi) decreased exosome generation thus suppressing CLL progression [100]. What is more, Poggio et al. successfully inhibited exosome production from prostate cancer cell lines by the deletion of nSMase2 or Rab27a via the CRISPR/Cas9 system, further modulating exosomal PD-L1 decrease to suppress cancer progression. More recently, Fan et al. [101] found that RAB22A could be predictive for MM relapse and be positively associated with exosome production and immune cell infiltration in MM patients. Si-RAB22A in MSCs impaired exosome secretion, further impeding their proliferative effects on MM cells [101]. However, given that the above manipulated targets are universal regulators for EV secretion, it is a non-specific inhibition of exosomes from both tumor and non-tumor cells. Therefore, it is necessary to investigate the potential negative effects on EV release in healthy cells. As non-specific inhibition might lead to off-target effects and unwanted side effects, future efforts should be devoted to elucidating different mechanisms of TDEV generation and their heterogeneous function for precise TDEV inhibition.

Furthermore, selected genomic mutations and signaling pathways are also associated with enhanced TDEV secretion. With the development of multi-omics and transcriptomics technologies, it is feasible to achieve target modifying TDEV release by regulating specific genes relevant to EV formation. In this regard, Tomofumi et al. [15] identified SORT1 and LAMP2 genes as two novel positive regulators associated with EV secretion, whose inhibition might reduce EV production and restore lenalidomide sensitivity. Ho et al. [102] found that HDAC3 knockdown (KD) in BMSC obviously reduced exosome release from a MM-BMSC co-culture model owing to the downregulation of TSG101, and further down-modified the expression of pro-survival miRNAs (such as miR380, miR382, miR15b, miR9986, and miR5191) in these exosomes, indicating the potential to inhibit MM progression via targeting MM-BMSC paracrine-autocrine signaling crosstalk. This work confirmed the efficiency of the HDAC3 inhibitor for MM therapy, hence its benefit for clinical transformation. Furthermore, several signaling pathways have also been found to contribute to modulating TDEV biogenesis and composition [96]. On such a basis, activated oncogenic signaling might be a possible candidate for the specific inhibition of TDEVs. For instance, glutamate signaling was reported to promote tumor-derived exosome formation, in which extracellular glutamate (Glu) transported by the Xc system activated glutamate metabotropic receptor 3 (GRM3) to positively regulate Rab27-mediated exosome production [103]. As a result, the inhibition of Glu antiporter system Xc-with sulfasalazine could suppress exosome release and its mediated crosstalk between MM cells and BMSCs, hence facilitating the combined therapeutic efficacy of BTZ and sulfasalazine [103].

### 4.2. Blocking EVs’ Uptake

Inhibiting the internalization of EVs by recipient cells is also a way to eliminate their mediated pro-tumorigenic effects. Of note, EV uptake is both selective and non-specific, where the interaction between EVs and target cells is the first step of EV uptake, which may be set to involve cell-specific mechanisms [104]. EV surface molecules’ mediated modes of interaction with different target cells play an important role in the process of EV binding and internalization [105]. However, current research on cell-targeted receptors on EVs is poorly defined. For instance, the cleavage of Bcl-xL caused by active exosomal caspase-3 is required for MM exosome to be internalized, which could be inhibited by a targeted chemical inhibitor or molecular mutant [106].

Although the detailed mechanisms for EV uptake are still unclear, endocytosis is recognized as a shared mode [107]. Targeting the classification of various endocytotic steps, researchers have invented corresponding pharmacological inhibitors [107,108]. In particular, Tu et al. [108] described the specific cellular mechanisms on the uptake of BMSC-derived EVs by MM cells by employing chemical inhibitors for distinct endocytosis routes or knockdown genes related to endocytosis with shRNA. Moreover, intracellular pro-survival pathways STAT1, STAT3, and ERK1/2 were also blocked when treated with chemical endocytosis inhibitors, and shRNA-targeting dynamin-2 preferentially facilitated the anti-MM effect of bortezomib both in vitro and in vivo. Additionally, antibodies, for instance heparin or its mimetic, specific to the Hep-II heparin-binding domain of fibronectin, could interfere with the binding of exosomes to target cells, which potently block the mediated pathogenic function [109].

### 4.3. Diminishing the Circulating EVs

Clinical translation has proved challenging because it is hard to target shared biochemical processes between normal and cancerous cells and this might cause undesired side effects. As a result, eliminating tumor-derived EVs can also be employed for cancer treatments [94]. Nao et al. [110] reported that anti-CD9 and anti-CD63 antibody treatment could be able to specifically target exosomes produced by human breast cancer cells to stimulate the clearance by macrophages, thus significantly reducing metastasis towards lymph nodes, lung, and the thorax. Based on this finding, it has been proposed that designing specific antibodies to target special molecules like proteins, glycan chains, and lipids on the surface of EV could stimulate the removal of cancer-derived EVs and subsequently inhibit their function [110]. Furthermore, designing the targeted removal of tumor-derived EVs based on physicochemical properties has proven promising.

Recently, Shin et al. [111] revised an α-helical (AH-D) peptide with a heightened α-helical structure and the ability to sense highly curved lipid membranes, especially exosomes derived from tumor cells (T-EXOs), and the peptide could target and induce T-EXOs’ rupture and degradation in a pH-enhanced manner associated with the tumor niche, which effectively decreases the exosomal PD-L1 level in the circulation and recovered function of CD8+ T-cells to synergistically enhance cancer immunotherapy. In this setting, although there are no relevant studies about the depletion of MM-EVs yet, the knowledge about MM-EV-specific antigenic molecules and characteristics might provide a new idea for specific antibody-targeted therapy to remove MM-EVs. Unfortunately, our current understanding of the physicochemical properties and molecular composition of MM-EVs is still a drop in the ocean. Furthermore, a recent study has tried to explain the functional role of MM-EVs in myeloma development and clinical applications from the perspective of mechanical characterization [112]. Single native EV from the fluid biopsies (BM, peripheral blood) of multiple myeloma and lymphoma patients were imaged using multiparametric AFM under aqueous conditions, thus qualitatively and visually elaborating and distinguishing the unique nanomechanical features (the elastic and viscous properties) of EVs, which are associated with their geometric features [112]. More specifically, the EVs in the BM have smaller Young’s moduli and viscous coefficients, as well as larger H/R values than those in blood [112].

## 5. EVs as Novel Drug Delivery Nanovectors

**Figure 3:**
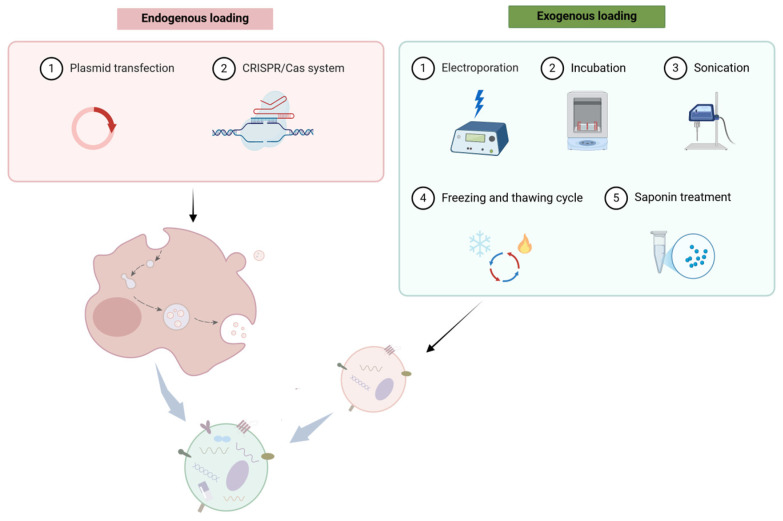
Strategies for loading cargoes into EVs. EVs can be modified for therapeutic applications by endogenous or exogenous methods. Endogenous strategies include transfection of donor cells by genetic modifications such as plasmid transfection and CRISPR-Cas9 system, thus regulating the secreted EV components prior to EV production. Exogenous strategies are based on the manipulation of isolated EVs by physical and chemical methods such as electroporation, incubation, sonication, freeze–thaw procedures, saponin treatment, and so on. Created in BioRender.com. Created in https://BioRender.com.

EVs have been evaluated as a promising therapeutic tool with an inherent advantage to deliver proteins, miRNAs, siRNAs, nanomaterials, and other therapeutic drugs, because of their superior biological features including nano-scale size, enhanced stability, excellent biocompatibility, low immunogenicity, no cytotoxicity, cell targeting, and tissue tropism [113,114]. It has been highlighted that EVs derived from most cell types (e.g., immune cells, tumor cells, MSCs) are robust and ideal delivery vehicles for therapeutic agents like miRNAs, siRNAs, and nanodrugs. Diverse methods have been employed for EV drug loading, which are mainly categorized into endogenous or exogenous approaches [115] (Figure 3). Different methods are suitable for carrying different drugs but also have their own advantages and disadvantages that need to be weighed. A great number of domestic and international studies of EV-based drug delivery systems (DDS) designed for cancer treatment have been assessed in a large array of clinical trials and have achieved encouraging results. For instance, the study of curcumin-loaded EVs for colorectal cancer therapy has entered phase I clinical trials (NCT01294072); the study of EV delivering siRNA or shRNA of the KRASG12D gene for pancreatic cancer treatment has also entered phase I clinical trials (NCT03608631).

Inspiringly, there have been several studies aimed at investigating the function of EVs, mainly exosomes as a carrier for the therapy of MM (Table 2). Zhang’s group generated the CACYBP siRNA-loaded exosomes via encapsulating CACYBP isoform2 siRNA into exosomes by direct electroporation inside the transfected parental ARP1 cells [30]. They further identified that CACYBP siRNA-loaded exosomes alleviated MM cell proliferation and OCs differentiation in vitro and exerted curative function on osteolysis in a PDX model in vivo [30]. Similarly, targeting the novel oncogene Aminoacyl-tRNA synthetase-interacting multifunctional protein 1 (AIMP1) with siAIMP1-loaded exosomes derived from OCI-MY5 cells could inhibit MM growth and bone lesion formation [116]. In their research, AIMP1 was reported to regulate histone H3 acetylation via interacting with ANP32A and promoted the proliferation of MM cells by the MAPK signaling pathway, cooperatively enhancing osteoclastogenesis with the modulation of RANKL to activate NFATc1 [116]. In particular, the commonality between the above two examples is the utilization of EVs from MM cells as DDS. One of its advantages is that tumor cell-derived EVs can preferentially target and be incorporated into homotypic cancer cells [117]. However, the presence of large amounts of carcinogenic components in tumor cell-derived EVs might limit their use in drug delivery [118]. In this regard, Zhou et al. [118] obtained tumor-derived EV membranes (TDEV membranes) by removing the contents in tumor-derived EVs, and then constructed innovative TDEV membrane-hybridized lipid nanovesicles (LEVs) via fusing TDEV membranes with phospholipids for precise delivery to tumors and the efficient transfection of siRNAs.

In a recent study, the vesicular secretome fraction (VSF) with synthetic miR-1252-5p was electroporated into EVs released by HEK293T cells, and these modified EVs were sufficient to increase the BTZ sensitivity of MM cells [119]. Mechanically, synthetic miR-1252-5p mimic decreased the expression of HPSE transcripts to inhibit MM cell viability and further potentiate the cytotoxic function of BTZ [119]. In addition, the group of Lombardi established a novel system to obtain miR-335-laden EVs induced in B cells (iEVs) by plasmid DNA induction [120]. The miR-335-laden B cell-derived iEVs decreased the expression of SOX4 and its downstream proteins including PTEN, AKT, and PI3K, thereby inducing apoptosis in MM cells [120]. Unexpectedly, miR-335-laden iEVs also heightened HIF1α expression in MM cells, but the reasons for this phenomenon and its subsequent effects remain to be further explored [120]. Of note, how to load therapeutic agents into EVs is a major obstacle to optimizing the EV-mediated DDS strategy. However, the conventional approaches have significant shortcomings, such as limited drug-loading efficiency, disrupted EV membrane integrity and stability, along with expensive costs for large-scale production. To address this problem, Yamayoshi et al. [121] designed a new approach by using anti-CD63 mAb-conjugated siRNA complexes with branched Arg linkers to capture exosomes, thus directly forming a DDS, and the carried siRNAs would suppress the targeted mRNA translation in the parental MM cells after the incorporation of these unique complexes, thus successfully altering the optimal molecules to exert therapeutic effects. What is more, a recent study utilized apoptotic vesicles (apoVs) from STS-induced apoptotic MSCs to establish BTZ/PC-apoVs with a synergistic therapeutic function, inheriting the anti-MM function of BTZ and apoVs [122]. As they reported, the synthesized BTZ/PC-apoVs could induce apoptosis in MM cells in vitro, whilst significantly ameliorating the inhibition of MM phenotypes and attenuating BTZ toxicity in vivo [122]. Furthermore, they found that Rab7 activation could elevate the production rate of PC-aopV from 33.1% to 47.6%, highlighting its potential regulator role for next-generation nanodrug-apoVs development [122].

In addition, it has been acknowledged that the ability to target specific cells is fundamental to the therapeutic role of EVs, which could be enhanced by modified surface proteins or other biomarkers [123,124]. B-cell maturation antigen (BCMA) is a novel and potent candidate for MM immunotherapy, which is expressed specifically on MM cells [125]. In this context, Yuan et al. [123] established a precise therapeutic delivery system by genetically engineered monocyte-derived exosomes with BCMA to endow remarkable cell specificity for malignant plasma cells. Then they successfully delivered BTZ to MM cells and exerted effective anti-tumor effects, meanwhile, without toxic side effects [123]. Furthermore, they suggested that anti-tumor function might be ascribed to the multiple functions of monocyte-derived exosomes in BM by regulating macrophage polarization, along with stimulating NK cell cytotoxicity and MSCs’ osteogenic transformation [123]. Similarly, He et al. generated reconstructed-BCMA nanovesicles (NVs) from the membrane of HEK-293T cells and verified their potential to target MM cells along with the synergistic anti-MM effects with a lower dose of BTZ mechanically via the remained capacity of BCMA to conjugate with APRIL/BAFF [126].

**Table 2:** EVs as novel drug-delivery nanovectors for MM therapy.

Cell Origin	Carried/Loaded Molecules	Loading Methods	In Vitro	In Vivo	References
MM cell lines ARP1	CACYBP siRNA	Electroporation	Suppressed MM cell growth	Improved the BM microenvironment and reduced bone erosion	[30]
OCI-MY5 cells	AIMP1 siRNA	Electroporation	Inhibited MM cell proliferation and osteoclast differentiation	Suppressed MM progression and alleviated bone destruction	[116]
HEK293T cells	miR-1252-5p	Electroporation	Decreased cell viability and enhanced BTZ sensitivity in MM cells	—	[119]
B cells	miR-335	Genetic modification	Stimulated MM cell apoptosis via regulating SOX4	—	[120]
MM cells	Anti-CD63 mAb-conjugated small interfering RNAs (siRNAs)	Chemical conjugation	Decreased levels of targeted mRNA transcripts in MM cell	—	[121]
Apoptotic MSCs	Biodegradable polycarbonate (PC) and bortezomib (BTZ)	Incubation	Induced MM cell poptosis	Ameliorated MM growth and bone injury, and improved the therapeutic efficacy of BTZ along with reduced side effects	[122]
human monocyte cell line THP-1	Antibodies targeting B-cell maturation antigen (anti-BCMA)	Genetic modification	Regulated macrophage polarization, and stimulated NK cells cytotoxicity as well as MSCs osteogenic transformation	Specifically targeted BTZ to myeloma, further inhibiting MM progression and MBD	[123]
HEK-293T cells	Re-BCMA	Genetic modification	Inhibited the MM cell proliferation and viability via blocking NF-κB pathway, and exerted a synergistic anti-MM function with BTZ in vitro	Presented a synergistic anti-MM function with BTZ	[126]

Consequently, it can be speculated that the anti-tumor ability of engineered EVs could be related to both the biological functionality of parental cells and modified molecules, which could provide more ideas for designing new targeted therapeutic regimens based on engineered EVs. To date, the powerful therapeutic effects demonstrated by multimodal synergistic therapy have been emphasized owing to the extremely complex pathophysiological mechanisms of cancer, while engineered EVs provide a new synergistic therapeutic paradigm by integrating EVs and functional nanoparticles, to organically combine various therapeutic approaches, such as immunotherapy, photothermal therapy, photodynamic therapy, or acoustic power therapy [124,127]. For instance, Xia et al. [128] developed engineered EVs secreted by HEK-293 with CDH17 nanobodies and indicated their potential as nanocarriers with tumor-targeting capability to load photothermal and chemo drugs, such as ICG or RRx-001, thus favoring photothermal therapy and targeted chemotherapy. Intriguingly, Xiao et al. [129] designed a biomimetic erythrocyte membrane-coated photothermal nanomissile BTZ@BPQDs@EM @anti-BCMA. The nanomissile successfully exhibited the promising cooperative effectiveness of chemotherapy and photothermal therapy owing to the carried BCMA antibody, photothermal therapeutic agent, and chemotherapy drug. Therefore, the integration of PTT inducers and chemotherapeutic agents into the nanoplatform system represented by EVs may be a promising strategy for the treatment of MM.

## 6. Engineered EVs as Antitumor Vaccines

TDEVs have become a hotspot for promising tumor vaccines, which is highly attributed to the carried immunostimulatory components with the potent capability to activate immune cells for more effective antitumor immunity, including tumor-associated antigens (TAAs), nucleic acids, and damage-associated molecular patterns [130]. Since natural tumor-derived EVs possess immunosuppressive properties able to trigger immune escape, potentially weakening the immunity of tumor-derived EVs, engineered modifications are needed to enhance their immunogenicity [124,130]. In general, there have been several modified strategies proposed to strengthen the precise immune responses mediated by TDEVs: (i) the enrichment of immunogenic molecules in TDEVs by directly modifying tumor cells; (ii) the conjugation of TDEVs with immunostimulators or adjuvants; and (iii) the loading of TDEVs into DCs [130]. In the context of MM, Xie’s team engineered MM exosomes to, respectively, upregulate the expression of IL-2, interferon-γ (IFN-g), and TNF-α, by the genetic manipulation of MM cells [131]. Comparative analysis revealed that the three exosomal vaccines had enhanced immunogenicity, and the exosomes released from the modified TNF-α-expressing MM cells especially caused an enhanced tumor-antigen-specific CD8+ T-cell response. Furthermore, they established MM cell exosomes presented with membrane-bound Hsp70 by the transfection of pcDNA_HSP70_ and found that it increased the expression of molecules such as lab, co-stimulatory molecules (CD40 and CD80), and inflammatory cytokines (IL-1β, TNF-α, Interferon-γ, and IL-12) [132]. Furthermore, they also demonstrated that the Hsp70-engineered exosomes from these MM cells could be used as a tumor vaccine to induce the maturation of DC and trigger the activation of NK cells, CD4 + Th1, and P1A-specific CD8+CTL, hence resulting in a more potent anti-tumor immunity [132].

To date, there exist several obstacles that certainly limit the search for effective cancer vaccines, especially limitations corresponding to the lack of potent tumor antigens able to elicit effective anti-tumor immunity. To address this, it has been suggested that the HLA-I ligandome of TDEVs might provide ideal targets for developing a novel and personalized cancer vaccine [133]. More recently, Kumar et al. [134] examined the presence of HLA-I ligandome, especially the tumor-relevant antigenic peptides of EVs from several cancer cell lines, MM cell line RPMI 8226 included. In particular, the peptide STAPPAHGV was identified in the EV ligandome of RPMI 8226 cells, known as a T-cell epitope of MUC1 characterized as an immunogenic tumor-associated antigen in several MM cell lines, as a possible source of adaption for potential cancer vaccines [134]. Furthermore, a better understanding of the interactions of TDEVs with various immune cells in the tumor immune microenvironment can help provide a basis for the development of new exosome-based biomarkers and effective cancer immunotherapy drugs [130]. Intriguingly, Malavasi et al. [135] found that microvesicles derived from MM cells exposed to CD38 antibody accumulated around immunoglobulin FcR+ immune cells and activated genes associated with the immune response of NK cell, despite the downregulation of cell cycle genes. Based on the above findings that CD38-EV can target specific immune cells, as well as parental MM cells with high expressions of CD38, CD38 can be used as a surface-modified molecule to confer its unique cellular targeting ability to engineered EVs, which could support the development of novel immunotherapies coupled to cytotoxic drug treatments. However, it has been reported that cell ectoenzymes contained in MM-MVs, including CD38, CD203a, CD73, CD157, and CD39, could co-responsibly drive the production of adenosine in the bone marrow microenvironment, further inhibiting T-cell function to achieve immunosuppression [41,136]. In this regard, a single modified target might be insufficient for effective anti-tumor effects; therefore, multitarget or combined peptide neoantigens should be taken into consideration for more potent and personalized immunotherapies. For instance, Cheng et al. [137] established genetically engineered multifunctional immune-modulating exosomes (GEMINI-Exos) carrying antibodies against human CD3, PD-1, OX40L, and EGFR on their surface, which successfully achieving powerful anticancer immunity via increasing CD8+ T cell infiltration and attenuating regulatory T-cell (Tregs) immunosuppression against EGFR-positive TNBC tumors.

At the same time, the lack of effective EV engineering approaches is another crucial bottleneck. Genetic manipulation and surface modification are two representative engineering approaches. The former is to target EV surface moieties through genetic engineering techniques, while the latter is attaching functionalized proteins based on the inherent properties of the vesicle membrane [130]. Traditional genetic engineering modification methods such as gene gun, electroporation, laser irradiation, and acoustic pore effect have the disadvantages of low transfection efficiency and susceptibility to external conditions. Therefore, the pursuit of more advanced technologies, like CRISPR-Cas genome editing systems and customized precise technology, has emerged [138]. Additionally, with the increasing unveiled details about EV protein-sorting, the integration of EV natural cargo-sorting mechanisms for EV cavity cargo loading and surface presentation has also gained attraction [139]. For instance, Chen et al. [140] designed an EV scaffold protein based on the ESCRT-dependent sorting mechanism, named the Late domain-based exosomal antibody surface display platform (LEAP), which can assist in displaying antibodies targeting TAA onto the EV surface, thus achieving targeted delivery to tumor cells. In their study, the LEAP-assisted presentation of αCD3 and αPD-L1 antibodies on the EV’s surface effectively modulated T-cell anti-tumor immunity against PD-L1+ tumor cells. Furthermore, considering that the modification process may alter the content and composition of EVs, thereby impairing their endogenous properties and inducing immunogenicity, there is a need to develop new methods to construct EVs without affecting their biological properties and to broaden their therapeutic applications [114].

Vaccine immunotherapy against cancer including MM represents a promising research direction in the area of immunotherapy. Regrettably, the development of EV-based anti-tumor vaccines is still at a very early stage. More animal and clinical trials have to be performed to validate their antitumor efficiency. Although no clinical trials of EV-based tumor vaccines for MM have yet been conducted, there have been several successfully implemented clinical studies and trials of EV-based tumor vaccines for NSCLC, obstructive cancer, melanoma, colorectal cancer, and so on [124,141]. Most of these clinical studies are mainly in Phase I or Phase II, and none have yet entered Phase III. Only one Phase II clinical trial has been completed on the use of EVs from dendritic cells modified with tumor antigens to augment NK- and T-cell immune responses as maintenance immunotherapy, improving prognoses for NSCLC patients (NCT01159288) [142]. Despite this, increasing ongoing clinic trials could always keep us hopeful about the clinical translation of EV-mediated tumor vaccine therapies.

## 7. Future Perspectives and Conclusions

MM is marked by multi-focal genetic heterogeneity and complex molecular evolutionary dynamics [143]. This complexity highlights that the pressing need for effective treatments and durable responses in high-risk (HR) MM patients, characterized by short survival, rapid progression, and poor prognosis, remains unmet. The early identification of high-risk factors and sustained minimal residual disease (MRD) negativity are important for the effective management of MM [140,142]. Advancements in technology and therapeutic methods have led us to focus more on the genetic alterations and clonal diversity of multiple myeloma at the molecular level [144]. The multiparametric fingerprinting of TDEVs could offer a more sensitive and valuable alternative to BM aspirates, which integrates multiple aspects of MM characterization, diagnosis, and therapeutic monitoring, enhancing the longitudinal monitoring of minimal residual disease (MRD). Furthermore, it supports the development of dynamic, evolutionarily adapted treatment strategies tailored to each patient’s unique molecular profile and clinical characteristics. Although immunotherapies, including ASCT, CAR T-cell therapy, and vaccination approaches, becomes increasingly important for HRMM, challenges such as non-response, relapse, and immunotoxicity still persist. Accordingly, deciphering the predominant immunogenic features mediated by TDEVs in MM biology could provide valuable insights into designing more effective anti-tumor immunotherapeutic regimens and helping to re-establish immune balance and achieve long-lasting immune control of the disease are crucial for enhancing its effectiveness.

However, there is still a long way ahead for the clinical application of EVs in MM management. Firstly, a solid theoretical foundation laid by basic experiments is key to designing strategies for clinical application. Continued efforts are still needed to broaden the comprehensive knowledge of TDEVs in MM, including their biogenesis, physical and chemical properties, as well as their functions. Meantime, it needs to strategically seek out areas that dovetail with other major advances in bioengineering and cancer treatment. For instance, can TDEVs be exploited to combine with monoclonal antibodies or bispecific antibodies for MM treatment to propose an innovative immunotherapy-coupled cytotoxic drug therapy? Is it practical to construct personalized vaccines by loading neoantigens relevant to specific mutations in patients? How do we improve the drug-loading efficiency and targeting of EVs? How do we design specific engineered EVs that are targeted to kill the malignant cells without potential injury to other somatic cells?

Secondly, both basic research and the clinical application of EVs are technically constrained by the lack of standardized protocols involving sample collection, EV isolation and purification, characterization methods, and reporting metrics [145]. For EV-based biomarkers, this possibly contributes to limited reproducibility and inevitable inter-study variation. In addition, the difficulty in distinguishing between EV sources and subtypes is a formidable obstacle, due to their overlapping biophysical and biochemical properties. Additionally, several key issues have to be elucidated in order to translate EVs into clinical therapy, including cost-effective large-scale production and the quality testing of therapeutic EVs, engineering modifications, targeting recipient cells, routes of administration, biodistribution, potential toxicity, and effective doses, and so on.

Collectively, the concept of therapeutically harnessing TDEVs for MM is promising and attractive. We expect that EV-based biomarkers and therapeutic strategies would be integrated into real-time clinical decisions in the near future.
